# Supplementary figures and images for: Cell-free supernatants from cultures of lactic acid bacteria isolated from fermented grape as biocontrol against Salmonella Typhi and Salmonella Typhimurium virulence via autoinducer-2 and biofilm interference
Source: PeerJ. 2019 Aug 26;7:e7555. doi: 10.7717/peerj.7555 (PMC6715067; doi:10.7717/peerj.7555)

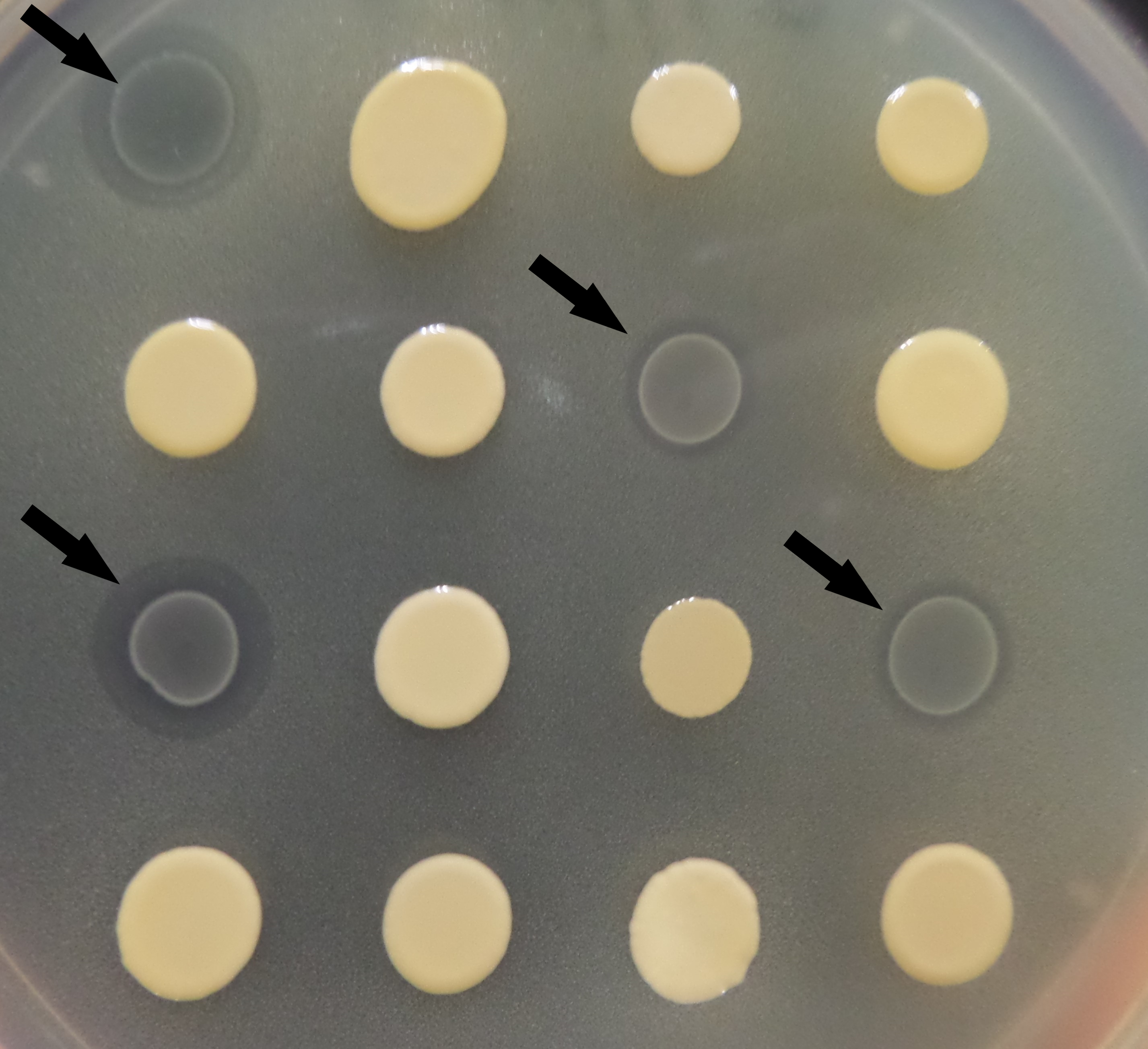

Supplement: Figure S1 — LAB colonies with inhibitory activity against Salmonella indicators are indicated by black arrow. [file peerj-07-7555-s006.png]

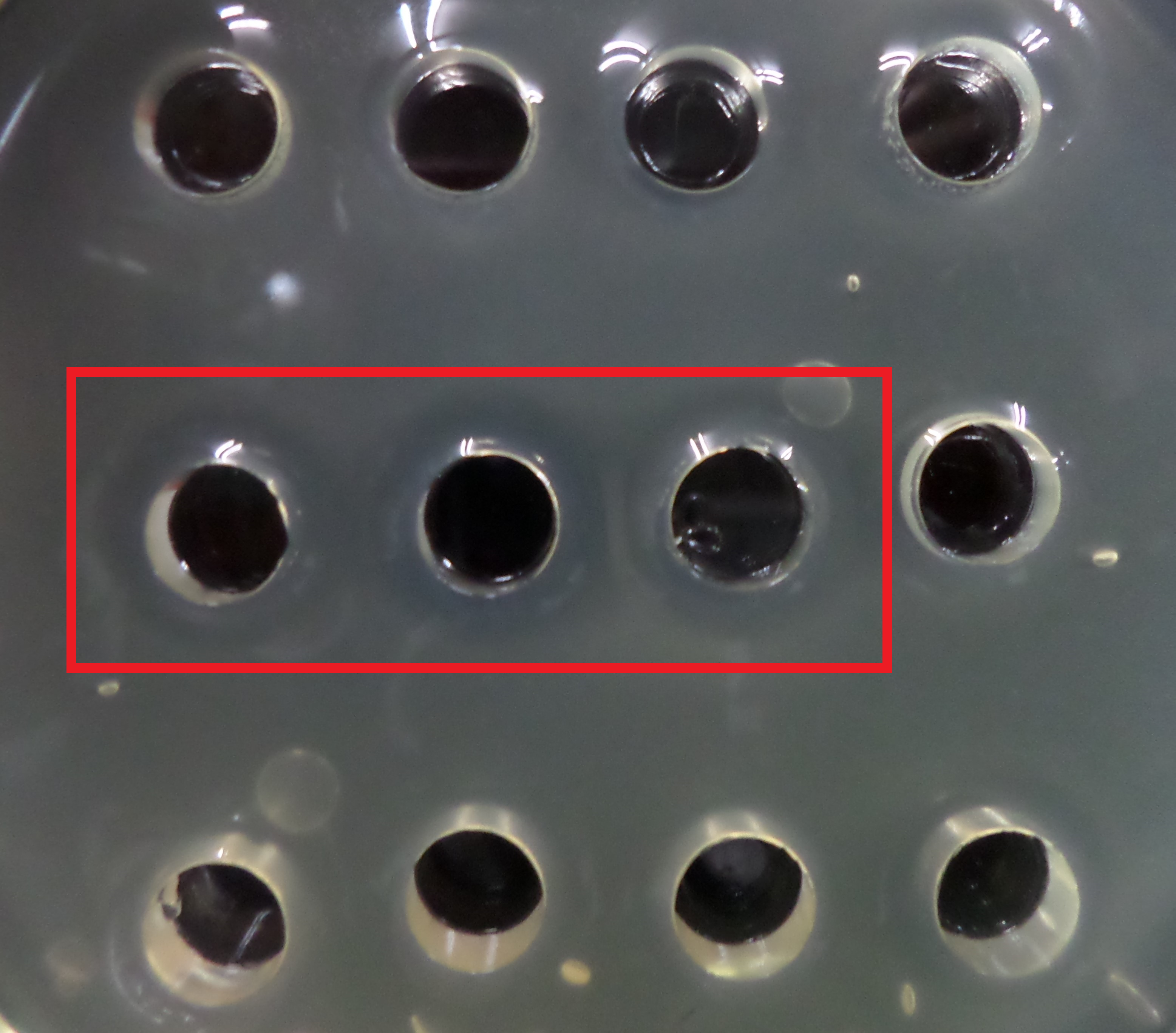

Supplement: Figure S2 — Red rectangle indicates the agar wells containing LAB-CFCS with inhibitory activity against Salmonella indicators. Bubbles are made by gas production of Salmonella indicators. [file peerj-07-7555-s007.png]

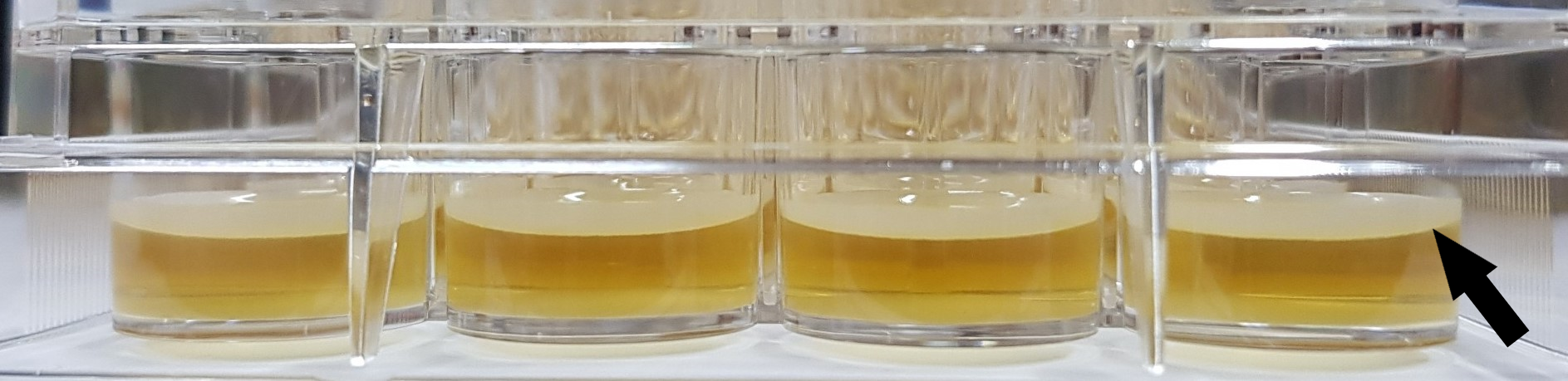

Supplement: Figure S3 — Black arrow indicates the biofilm layer of Salmonella indicators. [file peerj-07-7555-s008.png]

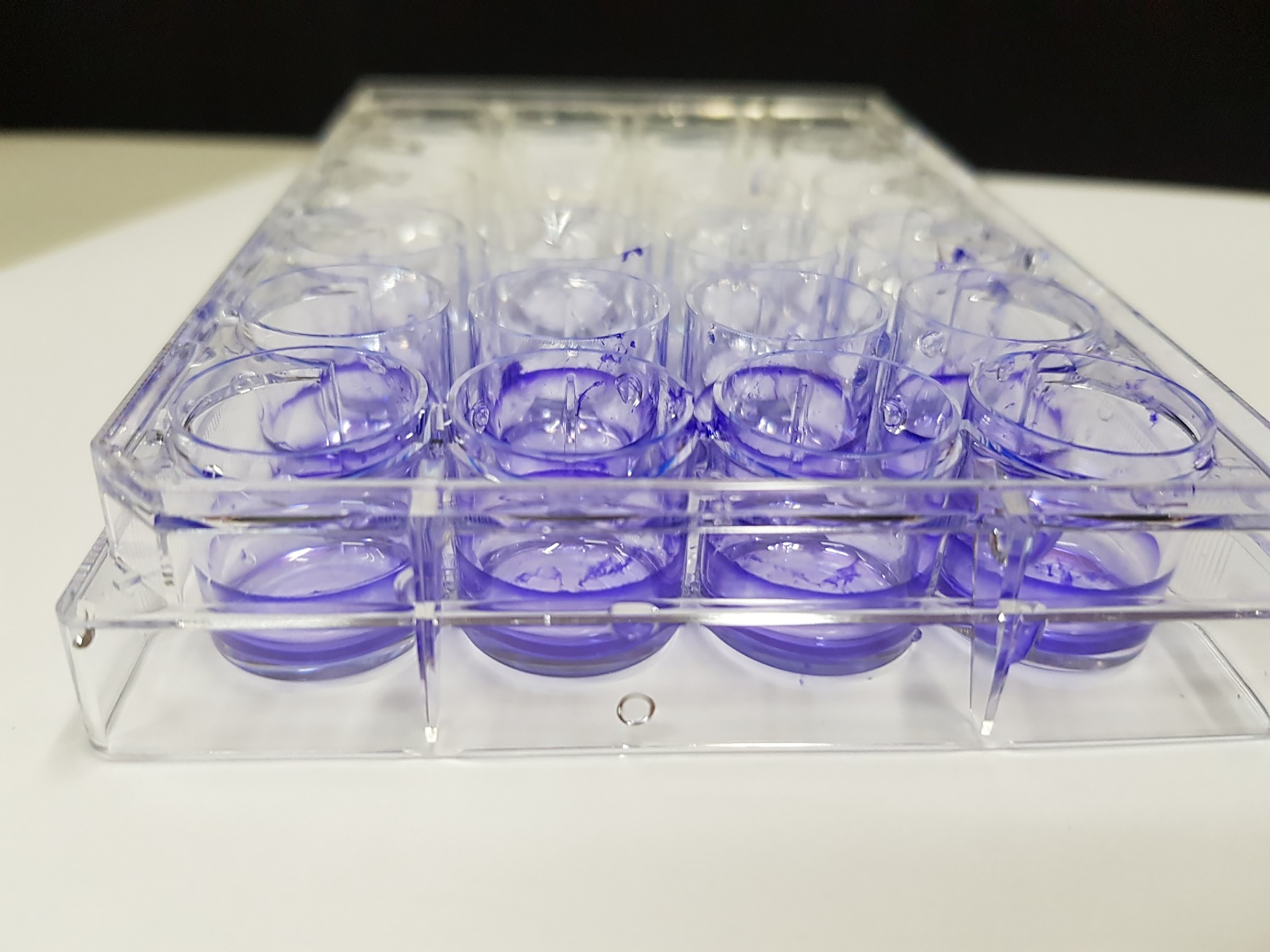

Supplement: Figure S4 — Microplate with biofilm after crystal violet staining. [file peerj-07-7555-s009.png]
